# Bioactivity of green-synthesized zinc oxide nanoparticles using *Fusarium oxysporum* extract on the expression of extended-spectrum beta-lactamase and biofilm-associated genes in the pathogen *Klebsiella pneumoniae*

**DOI:** 10.1016/j.jgeb.2025.100645

**Published:** 2025-12-28

**Authors:** Amani Kenaan Abd-Alrahman, Huda SA. AL-Hayanni

**Affiliations:** College of Science for Women, University of Baghdad, Baghdad, Iraq

**Keywords:** Biofilm, ESBL, *Fusarium oxysporum*, *Klebsiella pneumoniae*, Zinc oxide nanoparticles

## Abstract

Multidrug-resistant *Klebsiella pneumoniae* poses a serious clinical threat because of its ability to form biofilms and generate extended-spectrum beta-lactamase enzymes (ESBLs). This research investigated the influence of biosynthesized zinc oxide nanoparticles made from *Fusarium oxysporum* alcohol extract (FOE) on ESBL genes (*bla*TEM, *bla*CTX-M, *bla*SHV) and the biofilm-associated genes *mrk*A and *lux*S. The presence of the 16S rRNA, ESBL and biofilm genes was confirmed through subsequent polymerase chain reaction of the isolates. The FOE and zinc oxide nanoparticles both demonstrated significant antibacterial activity, with zinc oxide nanoparticles exhibiting greater inhibition with a minimum inhibitory concentration (MIC) of 26 μg/ml. Compared with untreated and FOE-treated isolates, those treated with sub-MIC concentrations of zinc oxide nanoparticles expressed significantly fewer ESBL and biofilm-related genes. The expression levels of the genes *bla*TEM, *bla*CTX-M, *bla*SHV, *mrk*A and *lux*S were downregulated below a ratio of 1.0 in each of the bacterial isolates. The biosynthesized zinc oxide nanoparticles demonstrated strong antibacterial and antibiofilm effects through the downregulation of bacterial antibiotic resistance and virulence genes in *K. pneumoniae*. The findings of this study demonstrate the ability of biosynthesized zinc oxide nanoparticles to function as a green and apotential alternative or support the role of antibiotcs for the treatment of multidrug-resistant (MDR) bacteria.

## 1 Introduction

Many harmful bacterial strains have developed resistance to one or more regularly used antibiotics, and some of these bacteria are resistant to more than one treatment. This has led to an enormous problem in world health: germs that are resistant to numerous drugs^1^. Conventional medicines have been less successful because of the increase in antibiotic-resistant microorganisms. As a result, there is an immediate need to create new approaches to control the spread of these pathogens and avoid going back to the days before antibiotics.^2^ Even in otherwise healthy people, the opportunistic pathogen *Klebsiella pneumoniae* can colonize their respiratory, urinary, and gastrointestinal systems. It causes a wide variety of infections, including pneumonia, abscesses, sepsis, diarrhea, urinary tract infections, wound infections, and severe respiratory disorders; it is the major cause of infections contracted while in the hospital. Infections caused by *Klebsiella pneumoniae* can cause serious health problems or even death if not treated effectively.^3^.Worldwide, reports of ESBL-producing strains dating back to the early 1980s indicate that the synthesis of these enzymes and AmpC β-lactamases are the primary mechanisms by which *K. pneumoniae* develops resistance to cephalosporins.^4^, ^5^ In regard to treating diseases caused by germs that have developed resistance, nanotechnology has shown encouraging results. Nanoparticles, which are defined as assemblies of materials with sizes ranging from 1 to 100 nm, are associated with distinct physical and chemical properties that enhance their antibacterial and antibiofilm properties. One of these properties particularly relevant to their nanoscale dimensions is the high ratio of surface area to volume.^6^, ^7^ Scientists investigated NPs as potential replacements for or additives to conventional antibiotics on the basis of their ability to bypass resistance mechanisms developed by the bacterium.^8^ The production of nanoparticles via fungi is a recently plausible biological approach, specifically, the use of *F. oxysporum*. Fungi can tolerate high loads of metals and efficiently concentrate them while also producing nanoparticles via enzymes of a controlled size and dispersion rate outside of the cell^9^, ^10^, ^11^. In general, the biological properties of zinc oxide nanoparticles (ZnO NPs) are useful in biomedical settings because of their lack of toxicity and eco-friendliness, and zinc compounds are considered safe by the FDA.^12^, ^13^ Recent studies have shown that zinc oxide nanoparticles (ZnO NPs) synthesized by *Fusarium oxysporum* exhibit efficacy against some extended-spectrum beta-lactamase enzymes (ESBLs)and (biofilm) genes expression in *Klebsiella pneumonia.*.^14^ One of the most critical mechanisms of antimicrobial action is the modulation of bacterial gene expression. Gene expression was measured via real-time PCR^15^: reported substantial downregulation of resistance and virulence genes following exposure to various nanoparticles.^15^, ^16^, ^17^, ^18^ On this basis, the objective of this study was to assess the gene expression associated with the beta-lactamase-encoding genes *bla*TEM, *bla*CTX-M, and *bla*SHV, as well as the *mrk*A and *lux*S genes associated with biofilm-related bacteria, in ESBL-producing *K. pneumoniae* exposed to *Fusarium oxysporum* alcoholic extract (FOE) and the biosynthesized ZnO nanoparticles there in.

## 2 Materials and methods

### 2.1 Investigated isolates

The current study consisted of the selection of five isolates of *Klebsiella pneumoniae* that were identified to produce biofilms and extended-spectrum beta-lactamases (ESBLs). All five isolates were taken from Iraqi patients admitted to Baghdad Medical City hospitals. The following descriptions and identifications of the isolates followed,^19^ who identified features, types, and macroscopic and biochemical tests of the five *Klebsiella pneumoniae* strains. Identification was confirmed again via the Vitek 2 system. Antibiotic susceptibility tests used the Vitek 2 system (to identify ESBL enzyme production) along with the double disc synergy method (DSM) for screening of ESBLs and for antibiotic resistance screening. The potential to produce biofilms of *Klebsiella pneumoniae* was measured via a 96-well microtiter plate assay via a crystal violet assay, as the assay was developed in.^15^ .

### 2.2 Genomic analysis of *Klebsiella pneumoniae* biofilm-associated genes, ESBL, and 16S rRNA genes

Genes related to biofilms (*mrk*A and *lux*S), ESBL resistance (*bla*SHV, *bla*CTX-M, and *bla*TEM), and the housekeeping gene 16S rRNA were amplified from genomic DNA isolated from *K. pneumoniae* isolates via polymerase chain reaction (PCR). Table 1 lists the names of the primers used in this investigation together with information about them, such as their sequence, annealing temperature, and predicted product size. PCRs were prepared following the manufacturer’s protocol (Promega, USA), as summarized in Table 2, Table 3**.** Following the method outlined by,^1^ we used a 100–1000 bp DNA ladder to determine the molecular size of the amplified products and resolved them via agarose gel electrophoresis (Clever, UK). Bands were visualized under a UV transilluminator (Clever, UK), and the presence of target genes was confirmed on the basis of the expected fragment sizes.

**Table 1:** Primer sequences of 16S rRNA, ESBL genes, and biofilm-associated genes.

Primer name	primer type	Primer’s sequence (5́____3́)	Product size (bp)	Reference
*16S rRNA*	F	TTGACGTTACCCGCAGAAGAA	187	Designed for the current study
	R	TCTACAAGACTCTAGCCTGCCA		
*mrk*A	F	ACGTCTCTAACTGCCAGGC	115	Designed for the current study
	R	TAGCCCTG TTGTTTGCTGGT		
*lux*S	F	TGTCGCGAAGAAAATGAACA	146	Designed for the current study
	R	TCAGATGATCGCGCATAAAG		
*bla* SHV	F	TGCGTTATATTCGCCTGTGT	146	Designed for the current study
	R	GCTGGCCAGATCCATTTCTA		
*bla*CTXـM	F	ACAGCAAAAACTTGCCGAAT	177	Designed for the current study
	R	TTCGGTTCGCTTTCACTTTT		
*bla* TEM	F	TTGCCGGGAAGCTAGAGTAA	188	Designed for the current study
	R	GAGGACCGAAGGAGCTAACC

**Table 2:** Preparation of the PCR mixture.

Components	Volume (μl)
Master Mix	12.5
Forward-primer (101 pmol/µl1)	11
Reverse-primer (101 pmol/µl1)	11
Nuclease Free Water1	6.51
DNA	4
Total volume	25

**Table 3:** PCR conditions for the genes.

Primer	Initial denaturation	Denaturation	Primer annealing	Elongation	Cycles	Final extension
16S rRNA	94 °C/5 min	94 °C/20 s	60 °C/45 s	72 °C/45 s	35	72 °C/10 min
*mrk*A	95 °C/5 min	95 °C/30 s	55 °C/45 s	72 °C/45 s	35	72 °C/7 min
*lux*S	95 °C/5 min	95 °C/30 s	55 °C/40 s	72 °C/60 s	35	72 °C/5 min
*bla*SHV	95 °C/5 min	95 °C/30 s	53 °C/40 s	72 °C/60 s	35	72 °C/5 min
*bla-*CTX-M	95 °C/5 min	95 °C/60 s	58 °C/55 s	72 °C/60 s	30	72 °C/5 min
*bla*TEM	95 °C/5 min	95 °C/30 s	60 °C/30 s	72 °C/60 s	35	72 °C/5 min

### 2.3 *Fusarium oxysporum* alcohol extract (FOE) preparation

Colony traits and microscopic characteristics facilitated the identification of a fungal isolate, *Fusarium oxysporum*, collected from the fungi laboratory in the department of biology at the college of science for women, University of Baghdad..^20^ The fungus collected was grown for 7 days on PDA media at a temperature of 30 °C, followed by freezing at 4 °C to prepare for future use in research.^21^. Mycelial discs were transferred to PGYP media to develop fungal biomass and incubated at 28 °C for 14 days. Following harvesting, washing, and population collection, the biomass was collected.^22^. The preparation of the extracts consisted of immersing the collected biomass in 80 % ethanol solution for 30 min with ultrasonication, allowing for 24 h of incubation, filtering through Whatman No. 1, and concentrating it with a rotary evaporator. The resulting extract was dried and stored at room temperature as a powder in sterile nylon bags.^23^ .

### 2.4 Biosynthesis of zinc oxide nanoparticles (ZnO NPs)

Zinc oxide nanoparticles (ZnO NPs) were synthesized via an alcoholic extract of *Fusarium oxysporum* via a modified method from.^24^ In brief, 10 mL of 6.25 % alcoholic fungal extract was added to a round-bottom flask containing 1000 mL of distilled water, which was subsequently heated with a stirrer. After pH neutralization, 17.5 g of zinc sulfate was introduced slowly, and sodium hydroxide was added. The mixture was poured into heat-resistant receptacles and placed in a furnace set to 200 °C for two hours. The precipitate was filtered, and the residue was washed thoroughly with distilled water and dried at 70 °C. The powder was collected and mixed with any finely powdered ingredients desired and stored in a sealed container for further use. Several analytical methods, including ultraviolet‒visible spectroscopy (UV‒Vis), Fourier transform infrared spectroscopy (FTIR), scanning electron microscopy (SEM), atmospheric absorption spectroscopy (AAS), energy dispersive X-ray spectroscopy (EDX), and zeta potential,^6^, ^7^ have been used to characterize the synthesized ZnO NPs.

### 2.5 Antibacterial activity of *F. oxysporum* alcoholic extract with zinc oxide nanoparticles as estimated from the resazurin-based microdilution method

The antimicrobial efficacy of FOE and ZnO NPs toward bacteria was assessed via resazurin-based microdilution in 96-well plates. Resazurin dye was used to measure bacterial growth, and the two agents were diluted serially. The blue wells indicate the minimum inhibitory concentration (no growth), whereas the pink or purple wells indicate bacterial growth. Sub-MIC values were defined as concentrations just below the MIC.^15^ .

### 2.6 Antibiofilm activity of alcoholic *F. oxysporum* extract and ZnO NPs

The antibiofilm activity was evaluated by microtiter plate method.^25^ with slight modifications. The bacterial suspensions were incubated in glucose-supplemented broth supplemented with either fungal extract or ZnO NPs at sub-MIC concentrations. After incubation, the wells were rinsed and dyed with crystal violet, and the dye was solubilized with ethanol. Biofilm inhibition was then quantified by measuring the OD at 580 nm compared with that of the untreated control.

### 2.7 Effects of *Fusarium oxysporum* alcoholic extract and biosynthesized zinc oxide nanoparticles on gene expression

- **RNA extraction and cDNA synthesis**

Five ESBL-producing *K. pneumoniae* isolates (KP1–KP5) that presented high resistance and biofilm formation ability were selected for gene expression analysis. Each isolate was inoculated in 3 mL of nutrient broth and treated with sub-MIC concentrations of either FOE or ZnO NPs. The cultures were incubated at 37 °C for 18–24 h. Total RNA was extracted via the TransZol Up Plus RNA Kit (TransGen Biotech, China) following the manufacturer’s protocol. The RNA purity and concentration were estimated via a NanoDrop spectrophotometer (Thermo Fisher, USA) at A260/A280, with values ​​approximately 2.0 considered acceptable.^26^ Complementary DNA (cDNA) was synthesized via the EasyScript® One-Step gDNA Removal and cDNA Synthesis SuperMix Kit (TransGen Biotech, China).

- **The primers:**

Primers targeting resistance genes (*bla*TEM, *bla*CTX-M, and *bla*SHV) and biofilm-associated genes (*mrk*A and *lux*S), which were specifically designed for the present study (Table 1), were obtained from Macrogen, Korea, and were prepared at a working concentration of 10 pmol/µL.

- **Quantitative real-time PCR (qRT‒PCR)**

qRT‒PCR was carried out in a 20 µL reaction mixture containing 10 µL of TransStart® Top Green qPCR Super Mix (TransGen Biotech, China), 3 µL of cDNA, 1 µL of each primer (forward or reverse), and 5 µL of nuclease-free water. The thermal cycling conditions included initial activation at 94 °C for 1 min, followed by 35–40 cycles of denaturation at 94 °C for 10 s, annealing at 58 °C for 15 s, and extension at 72 °C for 20 s.

- **Data analysis**

Relative gene expression levels were calculated via the ΔΔCt method.^27^, ^28^ A fold change > 1 indicated upregulation, whereas a value ​​<1 indicated downregulation.

### 2.8 Statistical analysis

All the data were analyzed via IBM SPSS version 25.0. The results are expressed as the means ± standard errors. Differences between groups were evaluated via one-way ANOVA followed by the least significant difference (LSD) post hoc test. Pearson’s correlation coefficient was used to assess relationships between parameters. Statistical significance was considered at p ≤ 0.05.^29^

## 3 Results and discussion

### 3.1 Molecular detection of ESBLs and biofilm-associated genes of *Klebsiella pneumoniae*

**Fig. 1:**
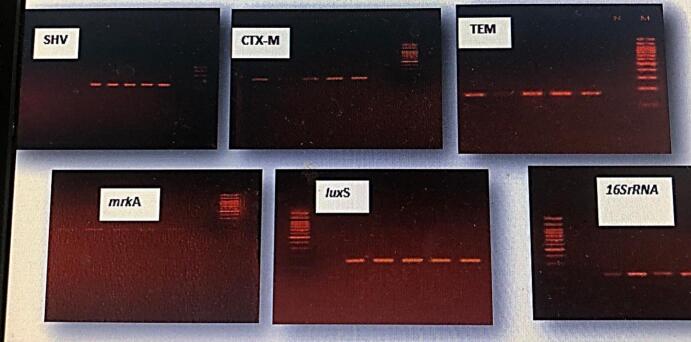
Agarose gel electrophoresis of amplified PCR products for the detection of 16S rRNA genes, ESBL genes, and biofilm-associated genes from *Klebsiella pneumoniae* isolates, a DNA ladder (M), *K. pneumoniae* isolates (Lanes 1–5), and 2 % agarose (60 min at 70 V) stained with ethidium bromide.

The detection of resistance genes (*bla*TEM, *bla*CTX-M, and *bla*SHV) and biofilm-associated genes (*mrk*A and *lux*S) was performed via the specific primers listed in Table 3. As illustrated in Fig. 1**,** all five ESBL-producing *K. pneumoniae* isolates were positive for the resistance genes *bla*TEM, *bla*SHV, and *bla*CTX-M, confirming their resistance to *β*-lactam antibiotics. In addition, screening for biofilm-associated genes revealed the presence of both *mrk*A and *lux*S in all the isolates, indicating their genetic potential for biofilm formation.

The current findings are consistent with those of,^30^ who reported that the majority of *K. pneumoniae* isolates from urinary tract infections harbored *bla*SHV genes, followed by *bla*TEM (55.8 %) and *bla*CTX-M (51.2 %). Similar observations were also reported by.^31^ and by,^1^ where *bla*SHV was the most prevalent resistance gene among clinical isolates. Sequence analysis further revealed no significant variation between Iraqi isolates and global strains, with 100 % similarity to many members of the Enterobacteriaceae family. Taken together, these results highlight the widespread dissemination of resistance determinants among *K. pneumoniae* isolates. The emergence of these genes is likely linked to the broad administration of antibiotics, which has contributed to the persistence and global spread of multidrug resistance.

### 3.2 Biosynthesis of zinc oxide nanoparticles (ZnO NPs)

**Fig. 2:**
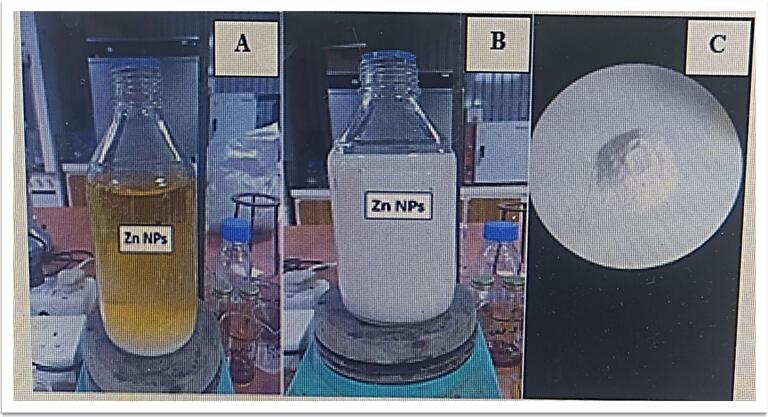
A, B, and C show the steps of manufacturing for the biosynthesis of zinc oxide nanoparticles (ZnO NPs). (A): Addition of alcoholic fungal extract, (B): deposition of ZnO NPs, (C) ZnO NP powder.

The biosynthesis of ZnO nanoparticles using *F. oxysporum* alcoholic extract **(**Fig. 2**)** was evidenced by a visible white precipitate and color change, which are common preliminary indicators of nanoparticle formation.^32^, ^33^ Subsequent characterization via techniques such as UV–Vis, FTIR, SEM, AFM, AAS, EDX, and Zeta potential confirmed the successful synthesis and stability of the ZnO NPs, which is consistent with previous findings.^25^, ^34^ .

### 3.3 Antibacterial activity of the *F. oxysporum* alcoholic extract and zinc oxide nanoparticles via the resazurin-based microdilution method.

The alcoholic extract of *F. oxysporum* demonstrated moderate antibacterial activity against ESBL-producing *K. pneumoniae*. The MIC values ​​were 3,906 μg/ml for four isolates (Kp1, Kp2, Kp3, and Kp5), whereas Kp4 required a higher concentration of 7,812 μg/ml, reflecting strain-dependent variability. The corresponding sub-MIC values ​​were 7,812 μg/ml for Kp1, Kp2, Kp3, and Kp5 and 15,625 μg/ml for Kp4 **(**Table 4**).**

**Table 4:** MIC and sub-MIC values of *F. oxysporum* alcoholic extracts and ZnO NPs against ESBL-producing *K. pneumoniae* isolates.

Isolates	MIC (μg/ml) Extract	sub-MIC (μg/ml) Extract	MIC (μg/ml) ZnO NPs	sub-MIC (μg/ml) ZnO NPs
Kp1	3,906	7,812	1,562	3,125
Kp2	3,906	7,812	1,562	3,125
Kp3	3,906	7,812	3,125	6,250
Kp4	7,812	15,625	195.3	3,906
Kp5	3,906	7,812	1,562	3,125

In contrast, the ZnO nanoparticles exhibited significantly greater antibacterial potency. The MICs ​​were 1,562 μg/ml for Kp1, Kp2, and Kp5; 3,125 μg/ml for Kp3; and 195.3 μg/ml for Kp4. The sub-MIC values ​​were 3,125 μg/ml for Kp1, Kp2, and Kp5; 6,250 μg/ml for Kp3; and 3,906 μg/ml for Kp4. These findings clearly indicate that ZnO NPs exert stronger antibacterial effects than alcoholic fungal extracts do and provide a suitable basis for subsequent gene expression studies.

*Fusarium oxysporum* extracts have antibacterial effects because bioactive compounds such as phenols and flavonoids affect membranes and metabolism.^33^ Sensitivity varies among strains because of biofilms or resistance genes,^35^ and the extraction method and concentration influence activity.^36^

ZnO nanoparticles from *F. oxysporum* are strongly antibacterial against ESBL-producing *K. pneumoniae*, functioning via cell wall attachment, protein disruption, reactive oxygen species (ROS) production, and Zn^2+^ release.^37^, ^38^, ^14^ Green synthesis may increase the stability and activity of fungal bioactive compounds.^39^

### 3.4 Antibiofilm activity of alcoholic *Fusarium oxysporum* extracts and ZnO NPs

**Fig. 3:**
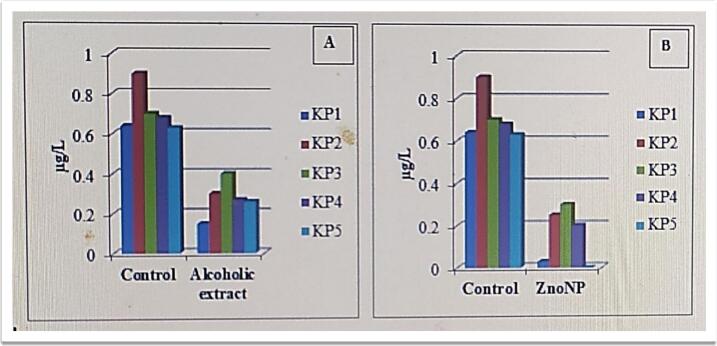
Anti-biofilm activity of (A) alcoholic fungal extract and (B) ZnO NPs against biofilm-forming, ESBL-producing *K. pneumoniae* isolates, as measured by a microtiter plate assay.

Treatment with both the fungal alcoholic extract and the ZnO NPs caused biofilm growth to decrease significantly. As shown in Fig. 3, the alcoholic extract had a moderate but consistent antibiofilm effect, with statistically significant inhibition observed in all the isolates. However, the biosynthesized ZnO NPs demonstrated a notably stronger inhibitory effect, with some isolates exhibiting near-complete suppression of biofilm formation.

The alcoholic extract reduces biofilm formation by blocking bacterial adhesion, quorum sensing, and matrix production.^40^, ^41^ ZnO nanoparticles further inhibit biofilms through ROS generation, surface interactions, and synergistic action with fungal compounds that disrupt adhesion, motility, and extracellular polymeric substances.^32^, ^42^, ^43^

### 3.5 Effect of *Fusarium oxysporum* alcoholic extract and biosynthesized zinc oxide nanoparticles on gene expression

**Fig. 4:**
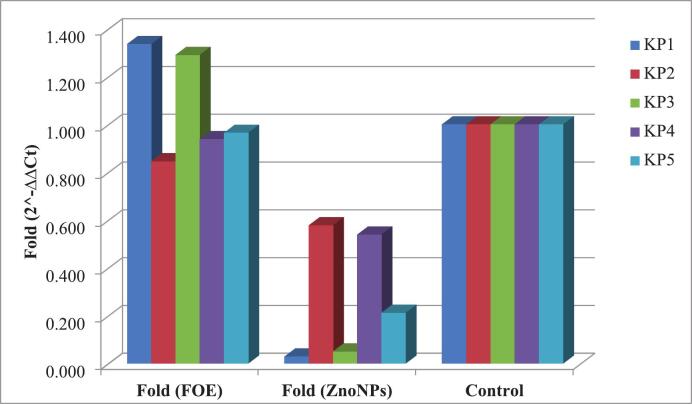
Effects of FOE and biosynthesized ZnO NPs on the *bla*TEM gene in ESBL-producing *Kp* isolates.

Five ESBL-producing *K. pneumoniae* strains (Kp1, Kp2, Kp3, Kp4, and Kp5), which previously demonstrated high levels of resistance and biofilm-forming ability, were selected to assess the impact of FOE and biosynthesized zinc oxide nanoparticles on the expression of resistance genes (*bla*TEM, *bla*CTX-M, and *bla*SHV) and biofilm-associated genes (*mrk*A and *lux*S). Quantitative real-time PCR analysis revealed that compared with FOE, the biosynthesized ZnO NPs had a more potent inhibitory effect on gene expression. Table 5 and Fig. 4 illustrate the changes in *bla*TEM gene expression across all the ESBL-producing KP isolates following treatment with FOE and ZnO NPs.

**Table 5:** Effects of FOE and the biosynthesized zinc oxide nanoparticles on the *bla*TEM gene in ESBL-producing *Kp.* isolates.

FOE						
*Kp* isolates No.	Control	*bla*TEM	H.K.G.	ΔCt	ΔΔCt	Fold (2^-ΔΔCt)
1	1.0	21.16	21.72	−0.560	−0.418	1.336
2	1.0	19.47	19.37	0.100	0.242	0.846
3	1.0	19.27	19.78	−0.510	−0.368	1.291
4	1.0	20.94	20.99	−0.050	0.092	0.938
5	1.0	19.45	19.54	−0.090	0.052	0.965
Mean ± SE		20.06 ± 0.41	20.28 ± 0.46	−0.22 ± 0.13	−0.080 ± 0.13	1.08 ± 0.10

ZnO NPs						

*Kp* isolates No.	Control	*bla*TEM	H.K.G.	ΔCt	ΔΔCt	Fold (2^-ΔΔCt)

1	1.0	25.57	20.59	4.980	5.122	0.029
2	1.0	21.07	20.42	0.650	0.792	0.578
3	1.0	23.29	19.1	4.190	4.332	0.050
4	1.0	21.97	21.22	0.750	0.892	0.539
5	1.0	21.88	19.78	2.100	2.242	0.211
Mean ± SE		22.756 ± 0.79	20.22 ± 0.36	2.53 ± 0.88	2.68 ± 0.88	0.28 ± 0.12
P value^#^		0.001*				

*Significant differences at probability p ≤ 0.05.

**Fig. 5:**
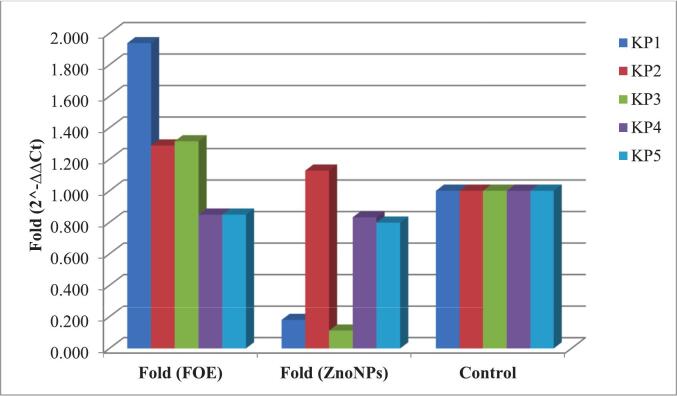
Effects of FOE and biosynthesized ZnO NPs on the *bla*SHV gene in ESBL-producing *Kp* isolates.

In terms of the *bla*SHV gene, compared with FOE, ZnO NPs significantly reduced gene expression in isolates *Kp*1 and Kp3, whereas in *Kp*2, Kp4, and *Kp*5, the difference between the two treatments was minimal. Overall, the mean fold change in *bla*SHV expression decreased from 1.24 to 0.61 following ZnO NP treatment **(**Table 6**,** Fig. 5**).**

**Table 6:** Effects of FOE and biosynthesized ZnO NPs on the *bla*SHV gene in ESBL-producing *Kp* isolates.

FOE						
*Kp* isolates No.	Control	*bla*SHV	H.K.G.	ΔCt	ΔΔCt	Fold (2^-ΔΔCt)
1	1.0	22.68	21.72	0.960	−0.954	1.937
2	1.0	20.92	19.37	1.550	−0.364	1.287
3	1.0	21.3	19.78	1.520	−0.394	1.314
4	1.0	23.14	20.99	2.150	0.236	0.849
5	1.0	21.69	19.54	2.150	0.236	0.849
Mean ± SE		21.95 ± 0.42	20.28 ± 0.46	1.67 ± 0.22	−0.25 ± 0.22	1.24 ± 20

ZnO NPs						

*Kp* isolates No.	Control	*bla*SHV	H.K.G.	ΔCt	ΔΔCt	Fold (2^-ΔΔCt)

1	1.0	24.99	20.59	4.400	2.486	0.179
2	1.0	22.16	20.42	1.740	−0.174	1.128
3	1.0	24.17	19.1	5.070	3.156	0.112
4	1.0	23.4	21.22	2.180	0.266	0.832
5	1.0	22.02	19.78	2.240	0.326	0.798
Mean ± SE		23.35 ± 0.57	20.22 ± 0.36	3.13 ± 0.67	1.21 ± 0.67	0.61 ± 20
P value^#^		0.50*				

*Significant differences at probability p ≤ 0.05.

**Fig. 6:**
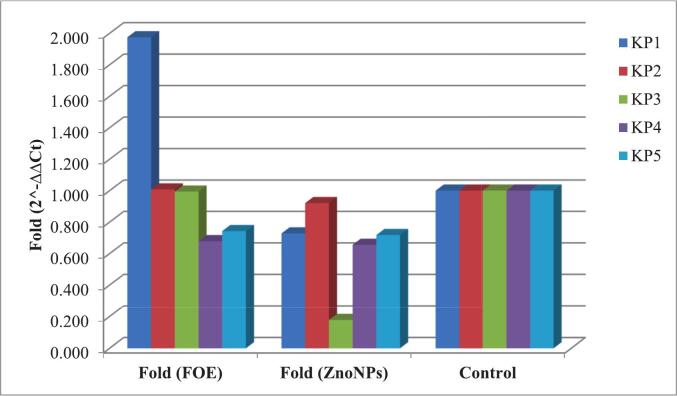
Effects of FOE and biosynthesized ZnO NPs on the *bla*CTX-M gene in ESBL-producing *Kp* isolates.

As shown in Table 7 and Fig. 6**,** compared with FOE, ZnO NPs also downregulated *bla*CTX-M expression in isolates *Kp*1, *Kp*2, and *Kp*3, while the effect was nearly similar to that of FOE in isolates *Kp*4 and *Kp*5.

**Table 7:** Effects of FOE and biosynthesized ZnO NPs on the *bla*CTX-M gene in ESBL-producing *Kp* isolates.

FOE						
*Kp* isolates No.	Control	*bla*CTX-M	H.K.G.	ΔCt	ΔΔCt	Fold (2^-ΔΔCt)
1	1.0	20.41	21.72	−1.310	−0.982	1.975
2	1.0	19.03	19.37	−0.340	−0.012	1.008
3	1.0	19.46	19.78	−0.320	0.008	0.994
4	1.0	21.22	20.99	0.230	0.558	0.679
5	1.0	19.64	19.54	0.100	0.428	0.743
Mean ± SE		19.95 ± 0.39	20.28 ± 0.46	−0.33 ± 0.27	0.00 ± 0.27	1.08 ± 23

ZnO NPs						

*Kp* isolates No.	Control	*bla*CTX-M	H.K.G.	ΔCt	ΔΔΔCt	Fold (2^-ΔΔCt)

1	1.0	20.72	20.59	0.130	0.458	0.728
2	1.0	20.21	20.42	−0.210	0.118	0.921
3	1.0	21.26	19.1	2.160	2.488	0.178
4	1.0	21.5	21.22	0.280	0.608	0.656
5	1.0	19.93	19.78	0.150	0.478	0.718
Mean ± SE		20.72 ± 0.28	20.22 ± 0.36	0.50 ± 0.42	0.83 ± 0.42	0.64 ± 0.12
P value^#^		0.135^ns^				

^ns^ Nonsignificant differences at probability p > 0.05.

**Fig. 7:**
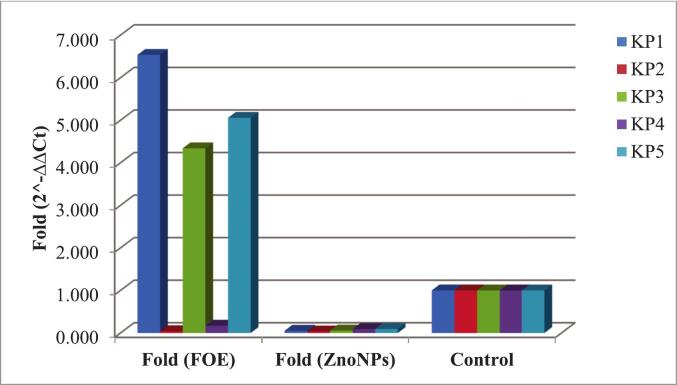
Effects of FOE and biosynthesized ZnO NPs on the *lux*S gene in ESBL-producing *Kp* isolates.

**Fig. 8:**
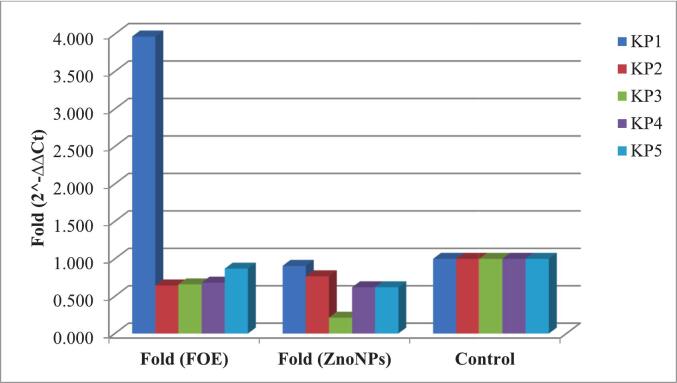
Effects of FOE and biosynthesized ZnO NPs on the *mrk*A gene in ESBL-producing *Kp* isolates.

With respect to biofilm-associated genes, both *lux*S and *mrk*A expression were reduced following treatment with ZnO NPs. Table 8 and Fig. 7 show that *lux*S expression was downregulated in all the isolates compared with FOE. Similarly, *mrk*A expression was downregulated in *Kp*1, *Kp*3, and *Kp*5 after ZnO NP treatment, while isolates *Kp*2 and *Kp*4 showed almost equal levels of regulation with both FOE and ZnO NPs (Table 9**,** Fig. 8).

**Table 8:** Effects of FOE and biosynthesized ZnO NPs on the *lux*S gene in ESBL-producing *Kp* isolates.

FOE						
*Kp* isolates No.	Control	*lux*S	H.K.G.	ΔCt	ΔΔCt	Fold (2^-ΔΔCt)
1	1.0	25.71	21.72	3.990	−2.708	6.534
2	1.0	30.69	19.37	11.320	4.622	0.041
3	1.0	24.36	19.78	4.580	−2.118	4.341
4	1.0	30.23	20.99	9.240	2.542	0.172
5	1.0	23.9	19.54	4.360	−2.338	5.056
Mean ± SE		26.98 ± 1.45	20.28 ± 0.46	6.70 ± 1.50	0.00 ± 1.50	3.23 ± 1.32

ZnO NPs						

*Kp* isolates No.	Control	*lux*S	H.K.G.	ΔCt	ΔΔCt	Fold (2^-ΔΔCt)

1	1.0	31.59	20.59	11.000	4.302	0.051
2	1.0	31.95	20.42	11.530	4.832	0.035
3	1.0	29.93	19.1	10.830	4.132	0.057
4	1.0	31.36	21.22	10.140	3.442	0.092
5	1.0	29.9	19.78	10.120	3.422	0.093
Mean ± SE		30.95 ± 0.43	20.22 ± 0.36	10.72 ± 0.27	4.03 ± 0.27	0.07 ± 0.12
P value^#^		0.044				

*Significant differences at probability p ≤ 0.05.

**Table 9:** Effects of FOE and biosynthesized ZnO NPs on the *mrk*A gene in ESBL-producing *Kp* isolates.

	FOE						
*Kp* isolates No.	Control	*mrk*A	H.K.G.		ΔCt	ΔΔCt	Fold (2^-ΔΔCt)
1	1.0	11.03	21.72		−10.690	−1.988	3.967
2	1.0	11.3	19.37		−8.070	0.632	0.645
3	1.0	11.68	19.78		−8.100	0.602	0.659
4	1.0	12.84	20.99		−8.150	0.552	0.682
5	1.0	11.04	19.54		−8.500	0.202	0.869
Mean ± SE		11.58 ± 0.34	20.28 ± 0.46		−8.702 ± 0.50	0.00 ± 0.50	1.36 ± 0.56

	ZnO NPs						
*Kp* isolates No.	Control	*mrk*A		H.K.G.	ΔCt	ΔΔCt	Fold (2^-ΔΔCt)

1	1.0	12.03		20.59	−8.560	0.142	0.906
2	1.0	12.1		20.42	−8.320	0.382	0.767
3	1.0	12.63		19.1	−6.470	2.232	0.213
4	1.0	13.21		21.22	−8.010	0.692	0.619
5	1.0	11.77		19.78	−8.010	0.692	0.619
Mean ± SE		12.35 ± 0.26		20.22 ± 0.36	−7.87 ± 0.37	0.83 ± 0.37	0.62 ± 0.12
P value^#^		0.296^ns^					

^ns^ Nonsignificant differences at probability p > 0.05.

Although the current research revealed a reduction in the expression of resistance genes (*bla*TEM, *bla*SHV, and *bla*CTX-M) and biofilm-associated genes *(mrk*A and *lux*S) in ESBL-producing *K. pneumoniae* following treatment with biosynthesized ZnO NPs, the available literature addressing the direct genetic effects of ZnO NPs on these specific genes is still scarce. Most published studies have focused primarily on the general antibacterial and antibiofilm mechanisms of ZnO NPs, including the generation of reactive oxygen species (ROS), the release of Zn^2+^ ions, and the disruption of cell membrane integrity.^44^, ^45^, ^46^

Evidence for gene-level regulation by ZnO NPs has been reported in other bacterial systems. For example,^47^ reported that ZnO NPs significantly downregulated the expression levels of all biofilm and virulence genes in *P. aeruginosa* clinical isolates. Additionally,^48^ reported that ZnO NPs suppressed the expression of capsule biosynthesis genes in *K. pneumoniae,* supporting the current observation that nanoparticles can directly modulate gene expression in clinically relevant pathogens. In addition, ZnO nanoparticles were discovered by.^25^ to play a role in inhibiting biofilm formation in multidrug-resistant *Proteus mirabilis* by severely downregulating *lux*S gene expression. These results align with our previous research on genes involved in biofilms, specifically *lux*S and *mrk*A, which play crucial roles in quorum sensing and fimbrial adherence. Similarly,^49^ reported that biosynthesized ZnO NPs were more efficient than chemically synthesized forms in reducing the expression of efflux pump genes in multidrug-resistant *Pseudomonas aeruginosa*, further supporting the superior efficacy of biogenic nanoparticles. These studies suggest that nanoparticles may interfere with bacterial regulatory pathways; however, no published data specifically address the modulation of *bla*TEM, *bla*SHV, *bla*CTX-M, *mrk*A, or *lux*S in *K. pneumoniae.*

The current results may therefore represent a novel contribution in this field, but they should be interpreted with caution. These findings strongly suggest that biosynthesized ZnO NPs can interfere with both antibiotic resistance and biofilm regulatory pathways in gram-negative bacteria. However, variations in the extent of downregulation across different isolates may reflect strain-specific genetic responses. Further molecular investigations are necessary to validate whether the observed downregulation is a direct genetic effect of ZnO NPs or a secondary consequence of their general antimicrobial activity. Future studies employing transcriptomic or proteomic approaches could provide more comprehensive insights into the mechanisms by which ZnO NPs influence resistance and the expression of biofilm-associated genes.

## 4 Conclusion

*Fusarium oxysporum* is a powerful synthetic agent for zinc oxide nanoparticles (ZnO NPs). Rather than using traditional antibiotics, fungal-mediated ZnO NPs could offer a greener alternative in the treatment of biofilm-related infections and multi-drug resistant (MDR) bacteria. ZnO NPs inhibitory effect was greater than that of the *Fusarium oxysporum* alcoholic extract. ZnO nanoparticles biosynthesized from *F. oxysporum* effectively reduced the expression of ESBLs and biofilm-associated genes in *K. pneumoniae* to below 1.0 ± 0.0, indicating strong antibacterial and antibiofilm activity, highlighting ZnO NPs as apotential alternative or support the role of antibiotcs against ESBL-producing *K. pneumoniae*.

## CRediT authorship contribution statement

**Amani Kenaan Abd-Alrahman:** . **Huda SA. AL-Hayanni:** Writing – review & editing, Supervision, Project administration, Methodology, Investigation.

## Declaration of competing interest

The authors declare that they have no known competing financial interests or personal relationships that could have appeared to influence the work reported in this paper.
